# Gut bacterial and fungal communities of the domesticated silkworm (*Bombyx mori*) and wild mulberry-feeding relatives

**DOI:** 10.1038/s41396-018-0174-1

**Published:** 2018-06-12

**Authors:** Bosheng Chen, Kaiqian Du, Chao Sun, Arunprasanna Vimalanathan, Xili Liang, Yong Li, Baohong Wang, Xingmeng Lu, Lanjuan Li, Yongqi Shao

**Affiliations:** 1 Institute of Sericulture and Apiculture, College of Animal Sciences, Zhejiang University, Hangzhou, China; 2 Analysis Center of Agrobiology and Environmental Sciences, Zhejiang University, Hangzhou, China; 3 Institute of Soil and Water Resources and Environmental Science, College of Environmental and Resource Sciences, Zhejiang University, Hangzhou, China; 4 National Collaborative Innovation Center for Diagnosis and Treatment of Infectious Diseases, State Key Laboratory for Diagnosis and Treatment of Infectious Diseases, the First Affiliated Hospital, School of Medicine, Zhejiang University, Hangzhou, China; 5 Key Laboratory for Molecular Animal Nutrition, Ministry of Education, Beijing, China

## Abstract

*Bombyx mori*, the domesticated silkworm, is of great importance as a silk producer and as a powerful experimental model for the basic and applied research. Similar to other animals, abundant microorganisms live inside the silkworm gut; however, surprisingly, the microbiota of this model insect has not been well characterized to date. Here, we comprehensively characterized the gut microbiota of the domesticated silkworm and its wild relatives. Comparative analyses with the mulberry-feeding moths *Acronicta major* and *Diaphania pyloalis* revealed a highly diverse but distinctive silkworm gut microbiota despite thousands of years of domestication, and stage-specific signatures in both total (DNA-based) and active (RNA-based) bacterial populations, dominated by the phyla Proteobacteria, Firmicutes, Actinobacteria, and Bacteroidetes. Most fungal sequences were assigned to the phyla Ascomycota and Basidiomycota. Environmental factors, including diet and human manipulation (egg production), likely influence the silkworm gut composition. Despite a lack of spatial variation along the gut, microbial community shifts were apparent between early instars and late instars, in concert with host developmental changes. Our results demonstrate that the gut microbiota of silkworms assembles into increasingly identical community throughout development, which differs greatly from those of other mulberry-feeding lepidopterans from the same niche, highlighting host-specific effects on microbial associations and the potential roles these communities play in host biology.

## Introduction

The silkworm *Bombyx mori*, a specialist herbivore that feeds on mulberry (*Morus alba*) leaves, is an economically important insect that has been domesticated for thousands of years to maximize silk fiber productivity [1, 2]. The importance of the silkworm is also reflected by its use as a powerful insect model for research due to its relatively large size and ease of rearing [3]. Especially the silkworm genome sequence was the first one available for Lepidoptera [4], the second largest insect order, which includes the most disruptive agricultural pests. These genome-based studies have facilitated our understanding of this species. For instance, genetic evidence has indicated that massive horizontal gene transfers have occurred from bacteria and fungi to *B. mori*, possibly increasing silkworm survival and fecundity [5] and suggesting the important role of microorganisms in the evolution of *B. mori*

With recent developments in the next-generation sequencing technology, a growing number of studies have cataloged and characterized microbial communities that live in diverse animal species, ranging from invertebrates to vertebrates. For example, a simple and distinctive gut microbiota, consisting of eight bacterial phylotypes, has been found in honey bees [6]. Three proposed enterotypes were identified by their enrichment in the human gut microbiome and were unrelated to nationality or host characteristics such as age or gender [7]. Furthermore, it is increasingly being recognized that these gut-inhabiting microbes are of crucial importance for the host, with effects that range from enhancing host metabolism to shaping the immune system [8–10]. For example, foregut bacteria in the herbivorous white-throated woodrat (*Neotoma albigula*) can degrade defensive plant secondary compounds, conferring detoxification potential to its host [11].

Like most animals, silkworms are associated with large consortia of nonpathogenic microbes [12]. Despite the existence of a large body of information on their biology, few studies have investigated the silkworm gut microbiota. Using 16S PCR-DGGE and clone library analyses, Xiang et al. estimated the midgut microbiota of two silkworm strains and identified 41 bacterial phylotypes [13]. By culturing methods, 11 isolates were obtained from the gut of the *B. mori* 5th-instar larvae, most of which have the capability of degrading various otherwise host-inaccessible polysaccharides from the diet [12], suggesting the potential importance of microbiota members for host nutrition and growth. These previous studies mainly yielded snapshots of the silkworm gut microbiome, as they were limited to relatively few sequences from samples pooled from multiple individuals or to a specific host life stage. Thus, the extent of the variation in this microbiota within individual silkworms, and the community dynamics across silkworm development, has been unclear. Furthermore, compared to its wild relatives that also exploit mulberry leaves as a food source, how the domestication process shapes gut microbial communities of mulberry silkworms is still unknown.

To establish an ecological and comparative baseline for experimental studies on the silkworm gut microbiome, here by using both culture-dependent techniques and culture-independent high-throughput sequencing of 16S and internal transcribed spacer (ITS) regions of the ribosomal RNA gene, we conducted a systematic investigation of the microbial communities associated with the domesticated silkworm *B. mori* (the standard strain p50) and related genera (the large dagger moth *Acronicta major* Bremer and the mulberry pyralid *Diaphania pyloalis* Walker) collected from a mulberry-planting field. As most studies on the gut microbiota have relied on DNA sequencing, it is unknown whether these identified organisms were truly active inside the host or were dormant or dead cells resulting from harsh gut conditions, such as the extremely alkaline gut pH in lepidopteran larvae [14]. In this study, we further employed rRNA-Seq to characterize the metabolically active fractions of the community. This method has been employed to study microbial communities from diverse environments [15–17], such as the larval gut of the wood-feeding beetle [18].

Together, these comparative and systematic analyses establish a framework for comprehensive studies of the *B. mori* gut microbiome and yield insights into the influence of microbial symbionts in this economically important and model insect.

## Materials and methods

### Sample collection and rearing

The *Bombyx mori* inbred strain p50 was provided by the Silkworm Germplasm Bank at the College of Animal Sciences, Zhejiang University, China. The large dagger moth *Acronicta major* Bremer and the mulberry pyralid *Diaphania pyloalis* Walker were collected from a mulberry-planting field (30°18′N, 120°04′E). All insects were reared under standard conditions (25 ± 1 °C, 70 ± 5% humidity with a photoperiod of 14 h of light and 10 h of dark) using fresh mulberry leaves collected from a greenhouse.

### Total nucleic acid extraction and reverse transcription

The gut tissue dissected from freeze-killed insects was weighed, directly transferred into a 2 mL sterile cryogenic vial sitting on ice. Tissues were homogenized in a Precellys-24 homogenizer (Bertin Technologies, Aixen, France) by 5000 rpm for 20 s. The egg sample comprised 100 eggs due to the limited material of a single egg. Foliage was collected from a mulberry-planting field, cut into 2–3 cm squares with sterilized instruments, and washed in the buffer (10 mM EDTA, 20 mM Tris, 0.012% Triton X-100, pH 7.8) for 30 min on a shaker. Phyllosphere microbes were collected by centrifuge. The total nucleic acid was extracted using the MasterPure™ Complete DNA and RNA Purification Kit (Epicentre, Madison, USA). Specifically, template-free water “blanks” were processed with the same DNA extraction and PCR amplification kits as the negative controls to avoid reagent contamination. To ensure efficient extraction, lysozyme (50 mg/mL) was added into the tissue and cell lysis solution, and gut homogenates or leaf washing were incubated at 37 °C for 40 min. The amount and quality of the total nucleic acid were measured by 2100 Bioanalyser (Agilent, Palo Alto, USA). The extracted RNA was further reverse transcribed to cDNA for sequencing according to the manufacturer’s instruments using random primers (GoScript™ Reverse Transcription System, Promega, Madison, USA).

### High-throughput sequencing and analysis

Fungi and bacteria were identified with universal primers (Supplementary Table S1) for detecting a broad range of microorganisms [19–21]. We included appropriate negative controls at all steps in PCR reactions. The ITS and 16S region were amplified in triplicate from DNA/cDNA preparations, and identical volumes were pooled for Illumina MiSeq sequencing (Majorbio, Shanghai, China) according to a standard protocol [22, 23]. 66 DNA and RNA (cDNA) samples were sequenced successfully: 37, 20 and 9 samples belonging to the experiment “bacterial diversity” (a single individual insect for each sample), ‘metabolically active members’ (a single individual insect for each sample) and “mycobiota” (three individuals of the same species pooled due to the low amount of fungi), respectively. In addition, five samples of the ‘bacterial diversity’ experiment were re-sequenced to investigate potential methodological biases.

High-quality reads were analyzed using both QIIME [24] and mothur [25] as detailed in the Supplementary information, which yielded a similar pattern of clustering and the same significant variables (Supplementary Figure S9), validating the robustness of our analysis. Operational taxonomic units (OTUs) were clustered at 97% similarity against the Greengenes and Unite database for bacteria and fungi, respectively [26, 27]. Because there are abundant chloroplasts of mulberry leaves in the gut contents of silkworm, reads representing chloroplast were removed prior to further analyses. As an additional quality control, OTUs that could not be assigned to a phylum were removed too.

### Diversity measures and statistical tests

For the OTU richness and community diversity analyses, rarefied OTU tables were generated to prevent the possible heterogeneity among samples due to number of sequences and calculate Chao and Ace estimators, and Shannon and Simpson indices, respectively. Values were compared using one-way ANOVA least significant difference (LSD) test, or the nonparametric Kruskal–Wallis test when the data were not normally distributed. In each case, type of test was stated before the *P* value, and *P* ≤ 0.05 was considered statistically significant. These analyses were performed with the Statistical Package for the Social Sciences, version 20.0 (SPSS, Chicago, USA). For group comparisons, Bray-Curtis dissimilarity metric and permutational multivariate analysis of variance (PERMANOVA) were performed by using the *vegdist* and *adonis* function from the *vegan* package in the R programming environment [28, 29]. Bray–Curtis distance matrices were constructed using rarefied OTU abundance table and visualized in principal coordinate analysis (PCoA). Taxonomic heat maps were clustered by the complete linkage method with the Bray–Curtis distance and generated by *ggplot2* [30].

### Data deposition

Raw sequencing data were deposited in the NCBI Short Read Archive (SRA) BioProject PRJNA377390 under the accession number SRP100894.

## Results

### Comparative assessment of the gut microbiota among the mulberry-feeding Lepidoptera larvae

The large dagger moth *Acronicta major* (Lepidoptera: Noctuidae) and the mulberry pyralid *Diaphania pyloalis* (Lepidoptera: Pyralididae) are representative natural feeders of mulberry leaves, which share feeding niches in mulberry-planting fields. Similar to *B. mori* (Lepidoptera: Bombycidae), the leaf-mining *D. pyloalis* is essentially monophagous, feeding on mulberry and close relatives, whereas *A. major* is a generalist herbivore.

**Fig. 1:**
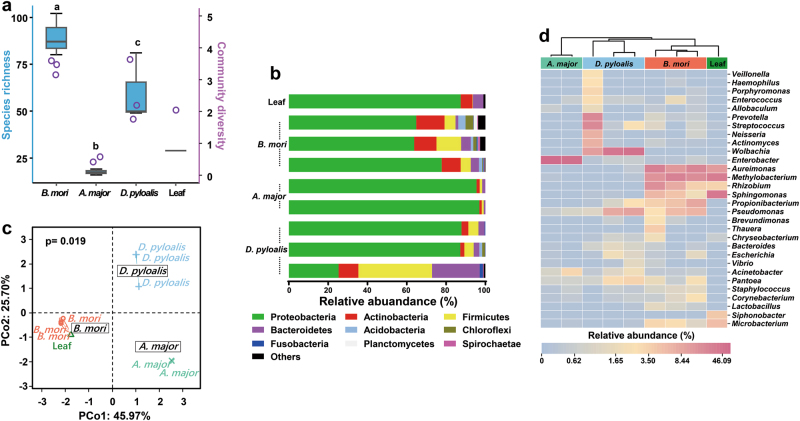
Gut bacterial community dynamics among mulberry-feeding lepidopterans. **a** Boxplot of species richness (number of OTUs) and community diversity measured by Shannon index. Letters above each host species indicate significant differences (one-way ANOVA, LSD post-hoc test, *P* < 0.05, see Supplementary Table S7) in the mean values. **b** Relative abundance of bacterial phyla in different samples. **c** PCoA plot based on community structure. Each symbol represents a sample, colored by host phylogeny. Variation in communities segregated strongly according to host phylogeny, with *B. mori*, *A. major* and *D. pyloalis* forming discrete groups (PERMANOVA test with 999 permutations, *P* ≤ 0.05, see Supplementary Table S8). **d** Heatmap showing the relative abundance of dominant taxa. Hierarchical cluster analysis was based on the Bray–Curtis distance with complete-linkage method. Each bar or column represents an individual insect

The whole gut of mature larvae (*B. mori*, 5th-instar; *A. major*, 6th-instar, and *D. pyloalis*, 5th-instar) was extracted to assess their associated microbial communities. The variability of community composition within individuals was first evaluated by using denaturing gradient gel electrophoresis (DGGE) of amplified 16S rRNA genes, a frequently used molecular technique for rapid fingerprint analysis of the microbial community [31]. DGGE profiles showed that at the same developmental stage there was little variation among different individuals of a population reared under identical environmental conditions (Supplementary Figures S1 and S2), a phenomenon widely observed in Lepidoptera [15, 32, 33]. Deep sequencing of these samples further revealed community diversity and composition. All of the cleaned sequences were clustered into OTUs that shared ≥97% sequence identity. Detailed sequence statistical information is shown in Supplementary Table S2. Chloroplast sequence contamination in 16S analyses is particularly problematic when sampling microbial communities in herbivorous insects [34]. After removing huge amounts of chloroplast sequences from original sequencing data, there were only hundreds of bacterial sequences left in some samples (6/59). However, rarefaction curves of Chao1 alpha diversity estimates still reflected a saturated sampling depth (Supplementary Figure S3). Good’s coverage, estimating what percent of the total species is represented in a sample, averaged 95.2% (Supplementary Table S3), suggesting that the majority of species in the gut were included in this study, as reported for insects [35]. Bacterial species richness and diversity were significantly different among the mulberry-feeding Lepidoptera larvae sampled (Fig. 1a). *B. mori* had the greatest diversity (90 unique OTUs per sample on average), and *A. major* had the lowest diversity (18 unique OTUs on average). This was also true for Chao and Ace richness estimators and Shannon and Simpson diversity indexes, which displayed similar patterns (Supplementary Table S3). Taxonomic analysis revealed that the most prevalent phylum was Proteobacteria, which, together with Firmicutes, Actinobacteria and Bacteroidetes, was detected in all samples (Fig. 1b). Overall, 12 OTUs were shared among all three groups regardless of species (Supplementary Table S4). The community dissimilarity was plotted based on Bray–Curtis (Fig. 1c) and UniFrac (Supplementary Figures S4a and b) metrics. PCoA ordination suggested that each host species group presented a distinct microbiota. In the scatter plot, the first two principal coordinates, PCO1 and PCO2, explained 45.97% and 25.70% of the data variation, respectively, clearly separating each group (PERMANOVA test with 999 permutations, *P* = 0.019). The microbiota of silkworms reared under controlled conditions clustered closely. Despite being collected from the field, *A. major* individuals shared microbiota with each other. A more diverse microbiome was observed in individuals of *D. pyloalis*; in one sample, as much as 60% of the reads belonged to Firmicutes and Bacteroidetes (Fig. 1b), but they grouped in the same cluster as well (Fig. 1c).

To visualize the distribution of different OTUs in host species groups, a heatmap was generated (Fig. 1d). A dendrogram was prepared using the Bray–Curtis index to compare the similarity of the bacterial communities between groups. Each column represents an individual insect, and columns were clustered according to the similarity of bacterial abundance profiles. Each row represents an OTU assigned to the genus level. While microbial population shifts in both abundant and less abundant genera were detected in individuals, we found that the samples still clustered based on host phylogeny, which harbored highly distinct microbial communities. Individuals of the same species were associated with a common bacterial community composed of the same dominant members. Only a limited set of microbial taxa, for example *Pseudomonas*, *Pantoea* and *Acinetobacter*, was present in all larvae. The bacterial diversity observed in *A. major* was remarkably simple, primarily being composed of *Enterobacter*. In contrast, abundant *Wolbachia* (40.60%) were present in *D. pyloalis*, and some bacteria, such as *Actinomyces* and *Prevotella*, varied within host species, which may represent transient microbes. Compared to its wild relatives, a highly diverse gut microbiota was found in *B. mori*, even under controlled laboratory rearing conditions. Among individual silkworms, *Aureimonas*, *Methylobacterium*, *Rhizobium*, *Sphingomonas*, *Propionibacterium*, and *Microbacterium* were the most common genera. *Sphingomonas*, *Aureimonas* and *Methylobacterium* were also the major components of the bacterial flora of mulberry leaves (Fig. 1d). But notably, in contrast to *B. mori*, very low abundances of these bacteria appeared in either *A. major* or *D. pyloalis*. Furthermore, several groups, including *Lactobacillus* and *Thauera*, were specifically associated with the domesticated silkworm.

**Fig. 2:**
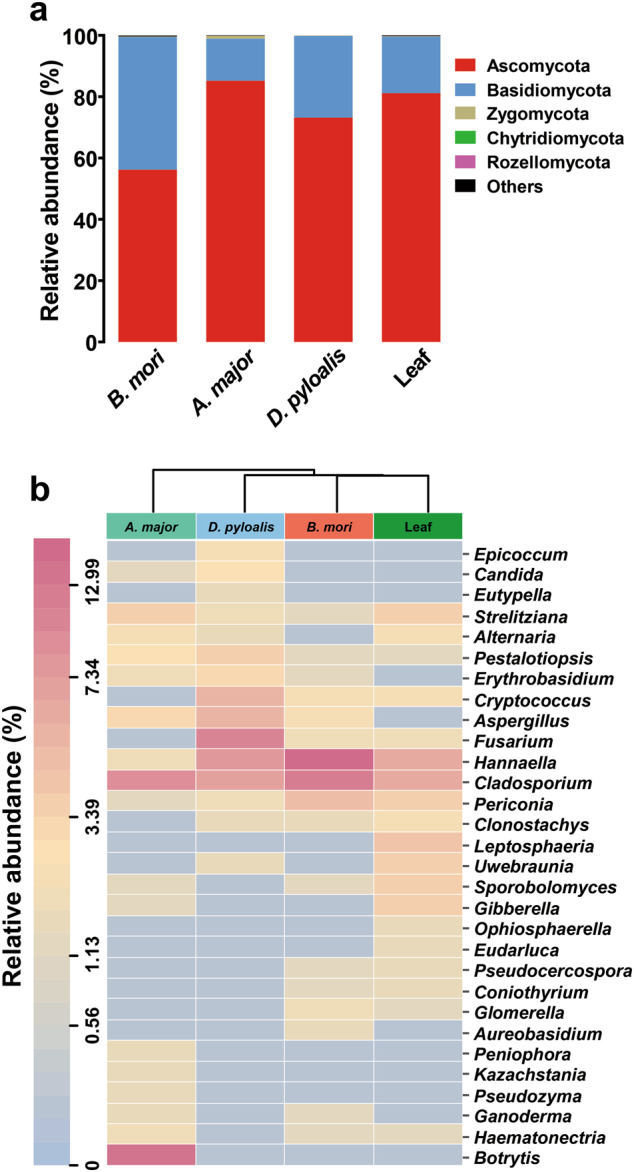
Gut fungal community dynamics among mulberry-feeding lepidopterans. **a** Relative abundances of fungal phyla in different samples. **b** Heatmap showing the relative abundance of dominant taxa in each species and mulberry leaves. Cluster analysis used the Bray–Curtis distance and complete-linkage method. Each bar or column corresponds to a species (three individuals for every species)

To examine fungal diversity, we further sequenced the ITS region of the rRNA genes derived from larval guts and their diet (mulberry leaves). Phylogenetic assignment of fungal sequences showed that two phyla, Ascomycota and Basidiomycota, collectively accounted for 99% of the fungal composition (Fig. 2a). At the genus level, *Cladosporium* and *Hannaella* dominated all samples. *A. major* harbored its own unique species, *Botrytis*. The mycobiotas of *B. mori* and *D. pyloalis* were more similar. Fungi, including *Fusarium*, *Cryptococcus* and *Clonostachys*, were specific for both species. Sequences assigned to *Aspergillus*, *Pestalotiopsis* and *Erythrobasidium* showed higher relative abundances in *D. pyloalis*, while *Periconia* was increased in *B. mori*. Interestingly, the sequences clustering within *Aureobasidium* were specifically found in *B. mori*. Most fungi associated with the larval gut were also identified as normal phyllosphere flora of the mulberry plant (Fig. 2b), indicating that wild mulberry plants could be reservoirs of diverse microbes. In contrast, the genera *Leptosphaeria*, *Ophiosphaerella,* and *Eudarluca* dominated the fungal community of the mulberry leaf, which were absent in insect guts.

### Spatial structuring of the *B. mori* gut microbiota

**Fig. 3:**
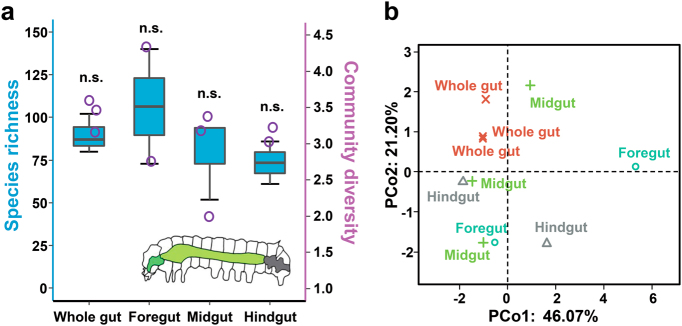
Spatial structuring of the *B. mori* gut microbiota. **a** Spatial variation of bacterial species richness and diversity. Inset: a schematic of the silkworm gut. n.s. not significant (one-way ANOVA, LSD post-hoc test, *P* > 0.05, see Supplementary Table S9). **b** PCoA plot showing variation in community structure among gut regions (PERMANOVA test with 999 permutations, *P* > 0.05, see Supplementary Table S8). Each point represents an individual insect

Like most lepidopteran larvae, the digestive tract of the silkworm is quite simple without any specialized structures, such as a proventriculus, and it accounts for the largest proportion of the body cavity, consisting of three regions: the foregut, midgut and hindgut [36]. The midgut represents the majority of the digestive tract, while the foregut and hindgut constitute smaller portions (Fig. 3a). The spatial segregation of microbes in this straight, tube-like gut was also analyzed. The foregut harbored a more diverse microbial community than the midgut and hindgut. On average, we observed 107 unique bacterial OTUs at the rarefied sequencing depth of 486 sequences per foregut sample, and 80 and 74 OTUs were present in the midgut and hindgut, respectively (Fig. 3a). PCoA showed that the microbiotas of different gut regions clustered separately (Fig. 3b, Supplementary Figure S5). A random distribution of the samples was found despite the midgut microbiotas being somewhat similar among individuals. The bacterial community profiles for each compartment are listed in Supplementary Figure S6. No clear pattern of localization within the digestive tract was observed. Thus, microbial communities were essentially indistinguishable between the foregut, midgut, and hindgut.

### Bacterial diversity across the life stages of *B. mori*

**Fig. 4:**
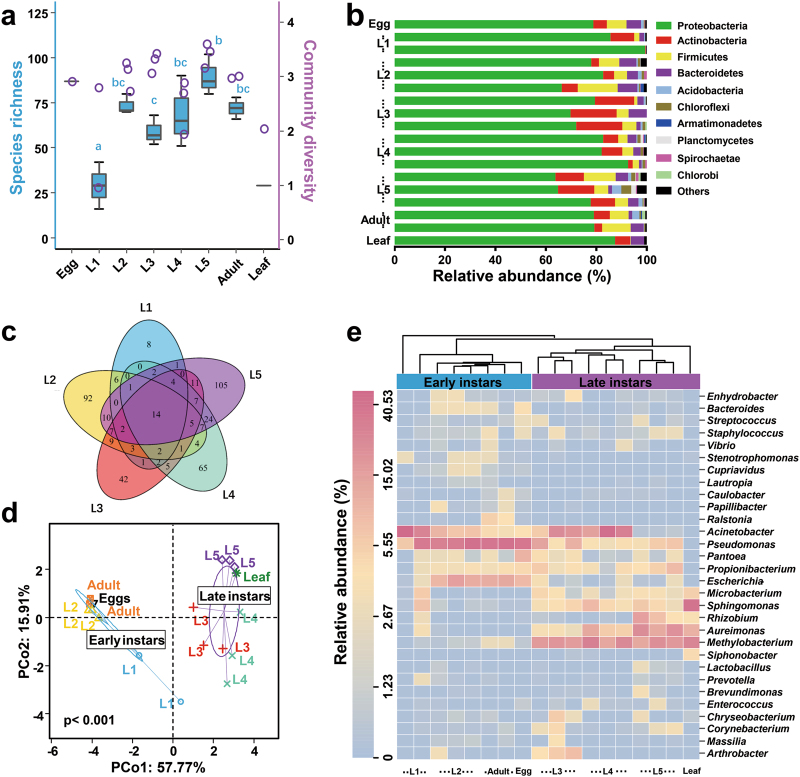
Gut bacteria dynamics across successive life stages of *B. mori*. **a** Boxplot of species richness and community diversity throughout development. Different letters indicate statistical significance (one-way ANOVA, LSD post-hoc test, *P* < 0.05, see Supplementary Table S10). **b** Phylum-level taxonomic succession. **c** Overlap of OTUs at the larval stage. **d** PCoA showing that gut microbiotas segregated strongly according to host developmental stage, with early instars and late instars forming discrete groups (PERMANOVA test with 999 permutations, *P* < 0.01, see Supplementary Table S8). **e** Heatmap of major taxa over the life cycle. Cluster analysis used the Bray–Curtis distance and complete-linkage method. Each point, bar or column represents a single individual

We next investigated variability of bacterial communities in relation to host development. Silkworms are holometabolous and undergo five larval instars (hereafter referred to as L1 through L5) before metamorphosing to short-lived reproductive adults (Supplementary Figure S7). We simultaneously sampled multiple individuals at each developmental stage over time. Our survey showed that the microbiota associated with eggs had high diversity, with 87 OTUs identified at this initial stage, while species richness decreased sharply in the 1st-instar larvae (Fig. 4a). The taxon number increased thereafter and reached a maximum in the 5th-instar larvae. After metamorphosis, bacterial species richness notably decreased again in the adults. Mulberry leaves were associated with a low number of species. Figure 4b illustrates the overall structure at the phylum level, with Proteobacteria, Actinobacteria, Bacteroidetes, and Firmicutes dominating the silkworm gut (96.67% of classified sequences). A Venn diagram analysis revealed that a subset of 14 bacterial taxa resided in the gut across the larval lifespan (Fig. 4c, Supplementary Table S5). A PCoA of the degree of bacterial community similarity showed relatively tight clustering of individuals from the same life stage, but differences between developmental stages were apparent (Fig. 4d, Supplementary Figures S4c and d). The data from the different larval instars were grouped into two clusters, 1st- and 2nd-instar larvae (hereafter referred to as early instars) and 3rd- to 5th-instar larvae (hereafter referred to as late instars). The bacterial communities of *B. mori* eggs and adults also clustered closely together with those of early-instar larvae. In contrast, the leaf sample grouped with the late-instar larvae. The first two axes explained >73% of the total variability in the PCoA. These results suggest a possible specialization of gut microbiota associated with host development in silkworms.

The corresponding heatmap of the OTUs assigned to the genus level offered a detailed view of the bacterial community composition (Fig. 4e). Across the 1st- and 2nd-instar larvae, clustering was not evident. One 1st-instar sample was separated from the others. However, during development, the gut microbiotas became increasingly similar to each other. Individual variation was smaller since the 2nd-instar (Homogeneity of molecular variance (HOMOVA) test, Supplementary Table S6). All larval samples fell into two clearly separate clades, specifically, the early instars and late instars. Early-instar larvae shared a similar profile of bacterial types as in eggs and adults, whereas the gut microbiota of late-instar larvae appeared closely related to that of the mulberry leaf. Most taxa, such as *Caulobacter* and *Ralstonia*, were only temporarily present in the gut and were likely transient bacteria. In contrast, *Staphylococcus*, *Enterococcus*, and *Microbacterium* represented consistently occurring bacterial taxa. *Pseudomonas* was the dominant genus (31.89%) in early developmental stages, followed by *Acinetobacter* (28.19%), *Escherichia* (7.78%), and *Propionibacterium* (1.93%). These bacteria were also found in the egg microbiota, likely acquired during the emergence from eggs. However, *Pseudomonas* and *Escherichia* decreased in late developmental stages, with a concomitant increase in the abundance of *Methylobacterium* (19.14%) and *Aureimonas* (9.67%). Therefore, bacterial community composition largely shifted between early and late instars.

### Metabolically active members within the gut

**Fig. 5:**
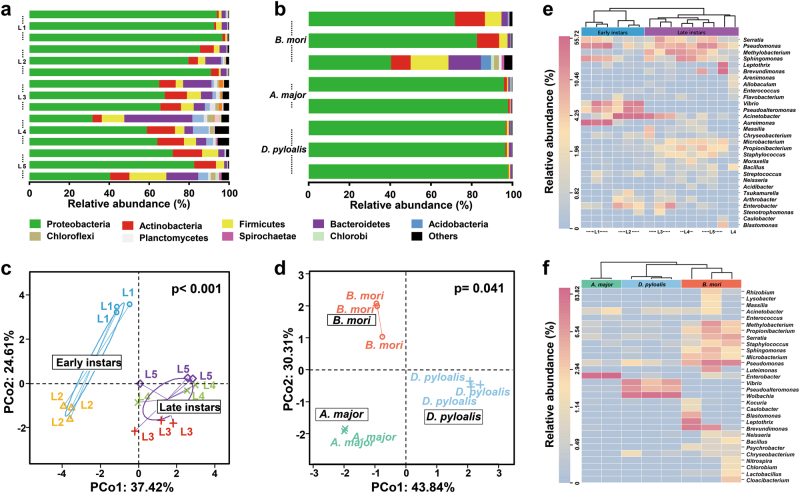
Metabolically active gut bacteria across life stages of *B. mori* and in *A. major*, and *D. pyloalis*. **a** Phylum-level community composition of *B. mori* and **b** other insects. **c** PCoA showing communities correlated with host developmental stage and **d** phylogeny (PERMANOVA test with 999 permutations, *P* ≤ 0.01, see Supplementary Table S8). **e** Heatmap of major taxa found in *B. mori* and **f** *A. major* and *D. pyloalis*. Cluster analysis used the Bray–Curtis distance and complete-linkage method. Each point, bar, or column represents a single individual

Given that the DNA-based approach used above allowed us to detect the presence but not activity of potential symbionts, we further determined the functional significance of these microorganisms within the larval gut using an RNA-based approach. We found that metabolically active bacteria also varied across life stages of the silkworm. Furthermore, a comparison of the active gut microbiotas of the domesticated *B. mori* and other mulberry-feeding Lepidoptera from the field revealed that each species had a characteristic pattern of active bacteria. Overall, 12 phyla were detected in the RNA fraction of the samples. Among them, the Proteobacteria, Firmicutes, Actinobacteria and Bacteroidetes were the dominant phyla, regardless of age or host species, but their ratios varied considerably among the groups (Fig. 5a, b). The phyla Bacteroidetes, Actinobacteria, and Firmicutes in the late-instar silkworms were abundant compared with other groups, with the vast majority of the sequences belonging to the Proteobacteria. PCoA results indicated that the silkworm gut microbiotas were divided into two subgroups, namely, early instars or late instars (the first two axes explaining 62% of the total variability), whereas individuals from each host species clustered exclusively (the first two axes explaining 74% of the total variability) (Fig. 5c, d, Supplementary Figure S8), consistent with the results of the DNA-level analyses (Figs. 1c and 4d).

Dendrograms were generated to further depict the composition and relatedness of bacterial communities from the larval gut (Fig. 5e, f). Some bacterial taxa were consistent colonizers of the silkworm gut, including *Pseudomonas* and *Acinetobacter* (Fig. 5e). Although a low abundance of *Serratia* was found in the DNA dataset, it was one of the dominant members of the RNA-derived fraction, indicating its high metabolic activity inside the gut. *Methylobacterium* and *Microbacterium* were also largely elevated in the late instars according to the RNA-based data. The increase in their metabolic activities was matched by increased abundances in the silkworm gut. In contrast, abundant *Aureimonas* (18.31%) were found in the DNA fraction (Fig. 4e), but they were not highly metabolically active (Fig. 5e). *Vibrio* and *Pseudoalteromonas* decreased in the RNA-based dataset of late instars. *Escherichia* and *Pantoea* did not show any activity (absent in the RNA data), which is typical for bacterial cell remnants or transient bacteria.

The comparative analysis also revealed distinctive patterns of active bacteria among *B. mori*, *A. major*, and *D. pyloalis*. Clustering analysis produced three clades, which clearly separated the domesticated silkworm from its wild relatives (Fig. 5f). Only a limited set of microbial taxa, including *Pseudomonas* and *Acinetobacter*, was shared among them. The within-group variation of the dominant bacterial taxa was small, showing a certain degree of consistency among individuals of the same species, even under field conditions. *A. major* harbored abundant and active *Enterobacter* (93.63%) in the gut. In samples from all other groups, only a small proportion of the sequences were associated with *Enterobacter*. Similarly, *Wolbachia*, *Vibrio*, and *Pseudoalteromonas* were the major active species in *D. pyloalis*, but they were absent from other samples.

## Discussion

Here, all surveyed insects including both domesticated and naturally occurring mulberry-feeding Lepidoptera, were intimately associated with diverse populations of microbes. Four phyla of bacteria (Proteobacteria, Firmicutes, Actinobacteria, and Bacteroidetes) dominated across life stages, similar to other insects [35, 37]. For the first time, the mycobiota of Lepidoptera, which primarily consisted of Ascomycota and Basidiomycota, was revealed by ITS sequencing. However, the fungal consortia were relatively small and inconsistently recovered. At the phylum level, there were no dramatic changes in the gut microbiota composition of the mulberry-feeding Lepidoptera. Spatial variation was not obvious along the gut, likely because the lepidopteran gut is simple [38], and the diet quickly passes through its gut [39].

We found that individuals of the same species assemble into essentially indistinguishable gut microbiota. Furthermore, a distinctive microbial community composed of a host-specific, taxonomically restricted set of microorganisms was identified for each species sampled, and no overlap of the most abundant bacterial taxa between host species was observed, suggesting a certain degree of host-species specificity. The microbial diversity of the insect gut is determined by various factors, including its environmental habitat, diet, developmental stage, and phylogeny of host [15, 35, 40]. Considering that the silkworms and wild-caught insects were reared under identical laboratory conditions until the final instars and that they ate mulberry leaves collected from the same source (DGGE profile revealed little variation among leaf microbiotas, see Supplementary Figure S2), it is highly likely that host phylogeny has a role in shaping microbial community structure within these mulberry-feeding Lepidoptera. In some animals, host genetics has been reported to play important roles in the selection of particular taxa [41].

Although *B. mori* has experienced intense artificial selection and has become completely dependent on humans for survival and reproduction, a greater diversity of bacteria was found in *B. mori* than in *A. major* and *D. pyloalis* taken directly from nature. The domestication process might adversely affect host defenses against nonindigenous microorganisms. For example, long-term captivity could impair or alter the development and activity of the immune system. In addition, sericulture is a labor intensive industry, where silkworm rearing and egg production occur under human manipulation, and microbes may be introduced during this process. A phylogenetically rich microbiota was found in silkworm egg masses. Finally, additional factors may also have been involved. Because of relative quantification in sequencing, a highly abundant microbe like *Wolbachi*a present in *D. pyloalis* is likely to lower community diversity.

Cluster analysis simplifies the dynamics of microbiotas into a limited number of variables that can be more easily visualized and manually inspected. We found a clear separation of the early (1st and 2nd)- and late (3rd to 5th)-instar gut microbiotas, and this pattern was consistent between total (DNA-based) and active (RNA-based) bacterial populations. Interestingly, previous studies have reported that there are two phases in the life of silkworms, namely the early (L1 and L2) and late (L3 and thereafter) larval instars [42, 43]. Therefore, shifts in the gut microbiota correlate with the changes in host developmental process, suggesting that the host’s physiology affects the microbiota composition. The exact mechanisms underlying the striking differences observed across development remain elusive but might include different niche-specific factors, such as gut oxygen levels, pH and microbe–microbe interactions [44]. In early instars, bacterial genera such as *Pseudomonas* and *Acinetobacter* are prevalent in silkworms, and their occurrence in Lepidoptera is well documented. For instance, *Acinetobacter* of field-collected *Helicoverpa armigera* larvae can metabolize insecticides, thus conferring host resistance to these compounds [45]. From the 3rd-instar, *Methylobacterium* and *Aureimonas* constitute a considerable and rather stable fraction of the gut microbiota; however, *Aureimonas* is not metabolically active. These leaf-associated bacteria are isolated from various plant species [46, 47] and were also found in our mulberry leaf microbiota, indicating that wild mulberry could be a reservoir of diverse gut microbes. Notably, *Methylobacterium* is abundantly found in other Lepidoptera, such as *Citheronia lobesis* and *Rothschildia lebeau* (Lepidoptera: Saturniidae) [48], and potentially contributes to nitrogen fixation in the gut. A Venn diagram revealed a shared community of 14 species found at all developmental stages, including *Enterococcus*, the most frequently detected bacteria in silkworms [13]. Symbionts in insects have been originally derived from free-living environmental microorganisms [49]. It is plausible that when new hosts (larvae) emerge, microbes are assembled into species-specific microbiotas from the environment, first from the egg and then from a native plant diet. Considering that silkworms are normally reared at high population densities, the transmission of microbes is also facilitated by specialized social contacts among host individuals, favoring the maintenance of microbial associations. This transmission strategy is reminiscent of the assembly of gut microbial communities in some other animals, including humans [50, 51].

*A. major* was associated with a much lower diversity of microbial taxa than *B. mori* and *D. pyloalis*. This phenomenon has been observed in other lepidopteran generalists as well [52]. Interestingly, the predominant fungus *Botrytis* has beneficial effects on the development and oviposition behavior of *Lobesia botrana* (Lepidoptera: Tortricidae) [53]. Abundant *Wolbachia* were present in the leaf-mining *D. pyloalis*. The endosymbiont *Wolbachia* is prevalent in insects, infecting 40–60% of all insect species, and they have long been studied for their complex interactions with the host, from manipulating host reproduction to conferring host resistance to viral infection [54]. All dominant bacteria were well represented in our RNA dataset, indicating a high protein synthesis potential and encompassing a large functional diversity that is worthy of future study. The genetic determinants of microbial colonization of the gut in different Lepidoptera species are also deserving of further study.

In conclusion, our results demonstrate that gut microbiotas of the domesticated silkworm greatly differ between early (L1 and L2) and late (L3 and thereafter) instars, and also differ from those of wild mulberry-feeding lepidopterans. Molecular and physiological mechanisms underlying gut symbiosis would be unraveled by making use of transcriptomic analyses of both the host gut tissue and its microbial community. Additional sampling of various silkworm strains would be required to assess the importance of the two-phase separation in *B. mori* biology and to see whether this is a species-wide effect. The well-established inventory of the microbiota of the silkworm, coupled with a large body of information on its genetics and physiology, makes *B. mori* a convenient model for evolution and ecology studies of host–microbe interactions. These findings will help improve silkworm economic traits, for instance by manipulating the gut microbiota to enhance its disease resistance ability and nutrient and energy utilization. On the other hand, advances in understanding the gut microbiota of these specialist and generalist herbivores may aid in the development of novel biocontrol strategies against notorious insect pests within the Lepidoptera.

## Electronic supplementary material

Supplementary materials -ys 201802
