# Supplementary material for: Gut bacterial and fungal communities of the domesticated silkworm (Bombyx mori) and wild mulberry-feeding relatives
Source: ISME J. 2018 Jun 12;12(9):2252–62. doi: 10.1038/s41396-018-0174-1 (PMC6092317; doi:10.1038/s41396-018-0174-1)
Supplement: Supplementary file 1 — Supplementary materials -ys 201802 [file 41396_2018_174_MOESM1_ESM.pdf]

Supplementary Materials

Title: Gut bacterial and fungal communities of the domesticated silkworm (*Bombyx mori*) and wild mulberry-feeding relatives

Bosheng Chen[1], Kaiqian Du[1], Chao Sun[2], Arunprasanna Vimalanathan[1], Xili Liang[1], Yong Li[3], Baohong Wang[4], Xingmeng Lu[1], Lanjuan Li[4] and Yongqi Shao[1,5,*]

[1]Institute of Sericulture and Apiculture, College of Animal Sciences, Zhejiang University, Hangzhou, China; [2]Analysis Center of Agrobiology and Environmental Sciences, Zhejiang University, Hangzhou, China; [3]Institute of Soil and Water Resources and Environmental Science, College of Environmental and Resource Sciences, Zhejiang University, Hangzhou, China; [4]National Collaborative Innovation Center for Diagnosis and Treatment of Infectious Diseases, State Key Laboratory for Diagnosis and Treatment of Infectious Diseases, the First Affiliated Hospital, School of Medicine, Zhejiang University, Hangzhou, China and [5]Key Laboratory for Molecular Animal Nutrition, Ministry of Education, China

[*]Correspondence: Yongqi Shao, E-mail: yshao@zju.edu.cn

Supplementary Methods

Denaturing gradient gel electrophoresis (DGGE)

DGGE investigation of microbiota profile of individual insect and mulberry leaf was performed along with the Illumina MiSeq sequencing (Takano *et al.* 2017, Thompson *et al.* 2008). To amplify the variable V6-V8 region of 16S rRNA gene, PCR was conducted with the primer set 968F-GC-Clamp and 1401R (Supplementary Table S1) under the following conditions: an initial cycle at 94 °C for 1 min; 25 cycles at 94 °C for 30 s, 55 °C for 30 s, and 72 °C for 1 min; and a final cycle of 72 °C for 10 min. The 50-μL PCR mixture contained 2× Gflex Buffer (TaKaRa Bio), 0.4 mM dNTP mixture, 0.2 μM each primer, 1.25 U of Tks Gflex DNA Polymerase, and 2.0 μL of DNA.

DGGE was carried out using the DCode Universal Mutation Detection System (Bio-Rad) onto the 8% polyacrylamide gel with a 20 to 80% denaturant gradient (100% denaturant = 7 M urea and 40% (v/v) deionized formamide). Gels were poured with the aid of a Bio-Rad gradient delivery system. PCR products (~300 ng) were electrophoresed in 1× TAE buffer (40 mM Tris, 20 mM acetic acid, and 1 mM EDTA) at 60 °C for 14 h at 100 V. Gels were stained with SYBR Green I (TaKaRa Bio) for 30 min and then photographed under UV light. DGGE profiles of bacterial 16S rRNA genes were analyzed using Quantity One software (version 4.6.2; Bio-Rad) and BioNumerics software (version 6.01; Applied Maths) as described previously (Chen *et al.* 2016, Lu *et al.* 2013, Thompson *et*

al. 2008).

Illumina sequencing

The V4-V5 region of the eubacteria 16S rRNA gene and the fungal nuclear ribosomal internal transcribed spacer (ITS) region were amplified with primers 515F-907R and ITS1F-ITS2, respectively (**Supplementary Table S1**). The sequencing primers contained the adapter and barcode sequences. The PCR reaction was performed in triplicate for each sample. For each reaction, 20 μL of mixture was prepared, including 5 x FastPfu reaction buffer, 250 μM dNTPs, 1 U FastPfu Polymerase (Transgene, Beijing, China), 200 nM of each primer (Majorbio, Shanghai, China), 1 μL of template and DNA-free water. The PCR reaction involved a single denaturation step at 95 ℃ for 3 min, followed by 27 cycles of 95 ℃ for 30 s, 55 ℃ for 30 s, 72 ℃ for 45 s, and finished after a final extension at 72ºC for 10 min. The triplicate reaction products were pooled and run on a 2% (w/v) agarose gel. Gel fragment of correct size was excised and purified with AxyPrep DNA gel extraction kit (Axygen, Union City, USA). After quantified by Quantifluor dsDNA system (Promega, Madison, USA), products were calculated into equal amount and mixed for Illumina MiSeq (San Diego, CA, USA) paired-end sequencing by a certified sequencing provider (Majorbio, Shanghai, China).

Quality filtering and de-multiplexing

The pair-end raw data were initially merged by FLASH (version 1.2.7) (Magoc and Salzberg 2011) with a minimum overlap of 10 base pairs. Merged sequences were trimmed and filtered by Trimmomatic (version 0.36) (Bolger *et al.* 2014). Sequences below quality score 20 were end-trimmed to remove low quality nucleotides, and subsequently a 50-nucleotide sliding window scanning from 5' end of read was used to trim group of bases lower than 20 quality score. Sequences lower than 50 bp, primer mismatch >2, barcode mismatch >0 were discarded. According to their unique barcode tags, sequences were demultiplexed for the OTU picking and taxonomic assignment.

OTU picking and taxonomic assignment

Sequences passed quality filters were initially taken to identify chimeric sequences by UCHIME algorithm in usearch (version 7.1) (Edgar 2010) with the script *identify_chimeric_seqs.py* implemented in the QIIME software (version 1.8.0) (Caporaso *et al.* 2010). The script *filter_fasta.py* was used for removing chimeric sequences. Afterwards, sequences were clustered into OTUs by usearch with the script *pick_otus.py* with a similarity threshold of 97%. Using the *pick_rep_set.py* script, the most abundant sequence in each OTU cluster was picked as the representative sequence. Representative sequences were classified taxonomically by the script *assign_taxonomy.py* using Greengenes (method, rdp; confidence, 0.80) and Unite database for bacteria and fungi respectively (DeSantis *et al.* 2006, Koljalg *et al.* 2013). To avoid the

affection of sequencing depth in difference samples, using the script *single_rarefaction.py* in QIIME, sequences from difference samples were rarefied to the same depth including a large portion of the OTUs and diversity (486 for bacteria DNA data, Good's coverage 94.1%; 612 for bacteria RNA data, Good's coverage 91.7%; and 177 for fungi DNA data, Good's coverage 86.2%), as reported for insects (Yun *et al.* 2014). Rarefaction curve and diversity indices (Sobs, Chao1, ACE, and Shannon) were calculated by 3 steps: First, rarefy OTU table by "multiple_rarefactions.py" with iteration=1000, step=60. The rarefied OTU table generated by random sampling at first step was used for calculating alpha diversity indices by script "alpha_diversity.py". Finally, results of every sample were concatenated into a single file by script "collate_alpha.py". Matrices of weighted and unweighted UniFrac were obtained from the script "beta_diversity.py" with the phylogenetic tree generate by "make_phylogeny.py".

Data visualization

1. The bar plots of relative abundance of bacteria and fungi in different host species and across different life stages of *B. mori* (Figures 1b, 2a, 4b, 5a and 5b) were visualized by GraphPad Prism (version 6.01) based on the rarefied OTU table, respectively.
2. The boxplot of bacteria gene copies (Figures 1a, 3a and 4a) was generated by R with the function *geom_boxplot* of the g*gplot2* package (R 2014, Wickham 2016).

3. Analysis of alpha diversity estimates and beta diversity indices (**Supplementary Table S3**) were based on the rarefied OTU table. Alpha diversity estimators Ace, Chao, beta diversity indices Shannon, Simpson, and Good’s coverage were computed by the *summary.single* command of the mothur software (version 1.30.1) (Schloss *et al.* 2009). The boxplots (Figures 1a, 3b and 4a) showing the species richness and community diversity were based on the OTU number and the Shannon index, respectively, and were visualized by the R package *ggplot2* with the function *geom_boxplot*.
4. Considering the large difference in taxon abundance between samples, the PCoA analyses (Figures 1c, 3b, 4d, 5c, 5d) were performed based on the relative abundances of OTUs, and the Bray-Curits distance was calculated using the R package *vegan* with the function *vegdist* and *procomp* (Kelly et al. 2015, Oksanen et al. 2007, Tang et al. 2016).
5. Heat maps (Figures 1d, 2b, 4e, 5e, 5f) were generated by the R package *vegan* with the function *vegdist* and *hclust*, and the *ggplot2* package with the function *geom_tile*. The Bray-Curits method was used to calculate the distance, followed with Complete-linkage clustering to visualize relationship between samples.
6. The Venn diagram (Figure 4c) was based on the OTU list (**Supplementary Table S5**), and generated by the R package *VennDiagram* with the function *venn.diagram*.

Mothur analysis

Demultiplexed sequences were introduced to Mothur software (Version 1.38.1) (Schloss *et al.* 2009). Sequences were joined into contigs by command “make.contigs” with a list file generated by command “make.file”. Any sequences with ambiguous bases (maxambig=0) and too-long sequences (maxlength=275) were removed by “screen.seqs” command. Afterwards, identical sequences were merged by “unique.seqs” command and aligned against to the Greengenes (Version 13_8) (McDonald *et al.* 2012) or SILVA (Release 128) reference database (Pruesse *et al.* 2007) respectively by “align.seqs” command (we have done both alignments to compare any differences between the two databases used). After removing gap characters (“filter.seqs” command) and duplicate sequences (re-run “unique.seqs” command), the remaining sequences were pre-clustered by command “pre.cluster” with diffs=2. “chimera.uchime” and “remove.seqs” commands were used for identification and removing of chimera sequences (Edgar 2010). The Naïve Bayesian method was used for sequence clustering (Wang *et al.* 2007), with Greengenes taxonomy as reference and a bootstrap value of 80% (“classify.seqs” command). Unknown sequences, Archaea sequences, chloroplast sequences, mitochondria sequences were removed by command “remove.lineage”. Cutoff=0.20 was used in the “dist.seqs” command before all sequences clustered into OTU at a divergence of 3% (“cluster.spilt”, taxlevel=4).

Phylogenetic tree was generated by FastTree (Version 2.1.3) with generalized time-reversible (GTR) substitution method (Price *et al.* 2010). Each classified OTU was assigned a taxonomy information with command “classify.otu” and binned into phylotypes with the “phylotype” command. For diversity analysis, all samples were normalized by “sub.sample” command to 447 sequences per DNA sample, and 697 sequences per RNA sample, in order to retain more samples. Alpha diversity analysis was performed by command “summary.single”, which generated observed species, Chao1, ace, Shannon indices of each sample. Rarefaction curves were obtained from the command “rarefaction.single”. Bray-Curtis distance matrices used in the heatmap and PCoA analysis were calculated by “dist.shared” command. UniFrac test based on phylogenetic tree was performed by command “unifrac.weighted” and “unifrac.unweighted”. To measure the variation within groups and significance of overall variability between groups, a nonparametric analog of Bartlett’s test for homogeneity of variance, Homogeneity of molecular variance (HOMOVA) (Schloss 2008), was performed by the command “homova”. Variation between different groups in PCoA analysis was calculated by PERMANOVA in R package “vegan”, using the function “adonis”, permutations=999 (Gilleland and Viii 2014).

The data produced by Mothur was further compared with that produced by QIIME, based on the OTU table combining the 30 most abundance genera in output of each software. PCoA analysis was based on Bray-Curtis matrices, p-

value was calculated by PERMANOVA with permutations=999.

Supplementary Tables

Supplementary Table S1 Universal primers for the identification of bacteria and fungi.

Primer/Probe	5ˋ modification	Sequence（5ˋ- 3ˋ）	Length（bp）	Target	References
27f		AGAGTTTGATCCTGGCTCAG	1465	Bacteria 16S rRNA gene	(Egert *et al.* 2005)
1492r		GGTTACCTTGTTACGACTT			
ITS1		TCCGTAGGTGAACCTGCGG	Around 700	Fungi ITS (internal transcription spacer) gene	(Anderson and Cairney 2004)
ITS4		TCCTCCGCTTATTGATATGC			
515F		GTGCCAGCMGCCGCGG	392	Bacteria 16S rRNA gene region for Illumina sequencing	(Xiong *et al.* 2012)
907R		CCGTCAATTCMTTTRAGTTT			
ITS1F		CTTGGTCATTTAGAGGAAGTAA	300	Fungi ITS gene region for Illumina sequencing	(Mukherjee *et al.* 2014)
ITS2		GCTGCGTTCTTCATCGATGC			
DB200-Qf		CGGYCCAGACTCCTACGGG	200	Quantitative RT PCR	(Lee *et al.* 1996)
DB200-Qr		TTACCGCGGCTGCTGGCAC			
Wolb-Qf		GCGAAGGCGTCTATCTGGTT	178	Quantitative RT PCR	(This study)
Wolb-Qr		AATCTTGCGACCGTAGTCCC			

968F-GC-Clamp		CGCCCGGGGCGCGCCCCGGG CGGGGCGGGGGCACGGGGGG AACGCGAAGAACCTTAC			
1401Ra		CGGTGTGTACAAGGCCCGGGA ACG	433	Eubacteria	(Shao *et al.* 2014)
1401Rb		CGGTGTGTACAAGACCCGGGA ACG			

Supplementary Table S2 Sequence statistics.

	Fungi (DNA-based)	Bacteria (DNA-based)	Bacteria (RNA-based)
**Amplified region**	ITS1F_2043R	515F_907R	515F_907R
**Sample number**	9	30	20
**Total bases (bp)**	287,133,960	749,122,552	438,107,448
**Passed QC (bp)**	175,515,614	561,640,440	345,957,709
**Rate passed QC (%)**	61.13	74.97	78.97
**Average Length (bp)**	306.86	396.47	396.4
**Total raw reads**	571,980	1,448,300	872,724
**Total chloroplast reads**	-	1,004,219	420,502
**Average reads**	63,553	48,277	43,636
**Minimum read count**	177	31387	29034
**Maximum read count**	22613	58626	54492
**Average reads (chloroplast & mitochondria sequence removed)**	-	14,803	11,488
**Minimum read count (chloroplast & mitochondria sequence removed)**	-	486	612
**Maximum read count (chloroplast & mitochondria sequence removed)**	-	50887	49473

Supplementary Table S3 Diversity metrics across various samples.

Sample ID	Reads	0.97					
		**OTU**	**Ace**	**chao**	**coverage**	**Shannon**	**Simpson**
B. mori-1	486	102	167 (137,223)	170 (135,243)	0.8971	3.59 (3.46,3.73)	0.0567 (0.047,0.0664)
B. mori-2	486	80	218 (174,282)	131 (103,193)	0.9156	3.15 (3.01,3.29)	0.091 (0.0763,0.1057)
B. mori-3	486	87	108 -97,132	100 -92,120	0.9465	3.46 (3.33,3.6)	0.0715 (0.0582,0.0848)
D.pyloalis-1	486	49	64 (55,87)	61 (53,88)	0.965	2.19 (2.03,2.35)	0.255 (0.22,0.2901)
D. pyloalis-2	486	50	65 (56,89)	61 (54,82)	0.9609	1.75 (1.58,1.93)	0.4392 (0.3863,0.4921)

D. pyloalis-3	486	81	100<br>-89,123	98<br>-87,125	0.9486	3.62<br>(3.51,3.73)	0.0441<br>(0.0375,0.0507)
A. major-1	486	19	40<br>(25,94)	41<br>-25,106	0.9753	0.41<br>(0.29,0.53)	0.8766<br>(0.8363,0.9168)
A. major-2	486	16	21<br>(17,39)	24<br>(17,59)	0.9877	0.58<br>(0.45,0.7)	0.8057<br>(0.7581,0.8532)
Leaf	486	29	122<br>-81,195	134<br>-68,310	0.9691	2.04<br>(1.92,2.15)	0.2078<br>(0.1839,0.2317)

Supplementary Table S4 Details of bacterial OTUs shared among *B. mori*, *A. major* and *D. pyloalis*.

OTU ID	Phylum	Genus
23	Proteobacteria	*Pseudomonas*
43	Proteobacteria	-
50	Firmicutes	*Staphylococcus*
177	Proteobacteria	*Pseudomonas*
435	Firmicutes	*Sporolactobacillus*
455	Actinobacteria	*Rothia*
486	Firmicutes	*Enterococcus*
520	Proteobacteria	*Acinetobacter*
681	Actinobacteria	*Corynebacterium*
714	Proteobacteria	*Escherichia-Shigella*
957	Firmicutes	*Allobaculum*
1068	Proteobacteria	*Pantoea*

Supplementary Table S5 Details of bacterial OTUs shared across the larval stages of *B. mori*.

OTU ID	Phylum	Genus
23	Proteobacteria	*Pseudomonas*
43	Proteobacteria	-
50	Firmicutes	*Staphylococcus*
131	Proteobacteria	*Sphingomonas*
224	Proteobacteria	*Acinetobacter*
270	Actinobacteria	*Rothia*
486	Firmicutes	*Enterococcus*
681	Actinobacteria	*Corynebacterium*
710	Actinobacteria	*Propionibacterium*
714	Proteobacteria	*Escherichia-Shigella*
724	Proteobacteria	-
806	Actinobacteria	*Microbacterium*
1039	Proteobacteria	*Pseudomonas*
1068	Proteobacteria	*Pantoea*

Supplementary Table S6 HOMOVA analysis across *B. mori* life stages.

Group	SSwithin/(Ni-1)	Compare to	p-value
L1	0.229134	L2	0.394
		L3	0.397
		L4	0.300
		L5	0.388
		adult	0.352
L2	0.073367	L3	0.104
		L4	0.106
		L5	0.115
		adult	0.791
L3	0.110096	L4	0.798
		L5	0.292
		adult	0.395
L4	0.101862	L5	> 0.999
		adult	0.397
L5	0.100954	adult	0.378
adult	0.0663201	-	-

Supplementary Table S7 Statistical test for species richness among different Lepidoptera species (one-way ANOVA, LSD post-hoc test).

Group1	Group2	Mean difference (I-J)	Std. Error.	Sig.	95% Confidence Interval	
					Lower Bound	Upper Bound
A. major	*B. mori*	-72.1667*	12.37717	.002**	-103.9832	-40.3501
	D. pyloalis	-42.5000*	12.37717	.019*	-74.3165	-10.6835
B. mori	*A. major*	72.1667*	12.37717	.002**	40.3501	103.9832
	D. pyloalis	29.6667*	11.07048	.044*	1.2091	58.1242
D. pyloalis	*A. major*	42.5000*	12.37717	.019*	10.6835	74.3165
	B. mori	-29.6667*	11.07048	.044*	-58.1242	-1.2091

*** : P≤ 0.05 , ** : P≤ 0.01**

Supplementary Table S8 PERMANOVA test based on distance matrices.

Test	Sample group	Value	Bray-Curtis	Weighted UniFrac	Unweighted UniFrac
PERMANOVA (“adonis")	RNA: *B. mori* early-instar vs. late-instar	R2	0.2959	0.29398	0.18967
		p	<0.001**	<0.001**	<0.001**
	RNA: domesticated silkworm vs. wild Lepidoptera insects	R2	0.401661	0.39017	0.3233
		p	0.041*	0.034*	0.011*
	DNA: *B. mori* early-instar vs. late-instar	R2	0.42206	0.41705	0.09134
		p	<0.001**	<0.001**	0.008**
	DNA: domesticated silkworm vs. wild Lepidoptera insects	R2	0.40079	0.28072	0.25725
		p	0.019**	0.1	0.028*

Foregut vs. Midgut	R2	0.24993	0.28198	0.20074
	p	0.4	0.3	0.9
Foregut vs. Hindgut	R2	0.34755	0.35081	0.51186
	p	0.6667	0.6667	0.1
Midgut vs. Hindgut	R2	0.28567	0.32796	0.24206
	p	0.4	0.3	1

Supplementary Table S9 Statistical test for species richness among different gut regions (one-way ANOVA, LSD post-hoc test).

Group1	Group2	Mean difference (I-J)	Std. Error.	Sig.	95% Confidence Interval	
					Lower Bound	Upper Bound
	Hindgut	33.0000	25.77359	.248	-30.0657	96.0657
Foregut	Midgut	26.5000	23.52796	.303	-31.0708	84.0708
	Whole gut	16.8333	23.52796	.501	-40.7375	74.4042
	Foregut	-33.0000	25.77359	.248	-96.0657	30.0657
Hindgut	Midgut	-6.5000	23.52796	.792	-64.0708	51.0708
	Whole gut	-16.1667	23.52796	.518	-73.7375	41.4042
	Foregut	-26.5000	23.52796	.303	-84.0708	31.0708
Midgut	Hindgut	6.5000	23.52796	.792	-51.0708	64.0708
	Whole gut	-9.6667	21.04405	.662	-61.1596	41.8263
	Foregut	-16.8333	23.52796	.501	-74.4042	40.7375
Whole gut	Hindgut	16.1667	23.52796	.518	-41.4042	73.7375
	Midgut	9.6667	21.04405	.662	-41.8263	61.1596

Supplementary Table S10 Statistical test for species richness across *B. mori* life stages (one-way ANOVA, LSD post-hoc test).

Group1	Group2	Mean difference (I-J)	Std. Error.	Sig.	95% Confidence Interval	
					Lower Bound	Upper Bound
L1	L2	-44.6667*	11.68332	.003**	-70.6987	-18.6346
	L3	-30.0000*	11.68332	.028*	-56.0321	-3.9679
	L4	-39.6667*	11.68332	.007**	-65.6987	-13.6346
	L5	-60.6667*	11.68332	.000**	-86.6987	-34.6346
	adult	-43.0000*	12.79844	.007**	-71.5167	-14.4833
L2	L1	44.6667*	11.68332	.003**	18.6346	70.6987
	L3	14.6667	10.44988	.191	-8.6171	37.9505
	L4	5.0000	10.44988	.643	-18.2838	28.2838
	L5	-16.0000	10.44988	.157	-39.2838	7.2838
	adult	1.6667	11.68332	.889	-24.3654	27.6987
L3	L1	30.0000*	11.68332	.028*	3.9679	56.0321
	L2	-14.6667	10.44988	.191	-37.9505	8.6171
	L4	-9.6667	10.44988	.377	-32.9505	13.6171
	L5	-30.6667*	10.44988	.015*	-53.9505	-7.3829
	adult	-13.0000	11.68332	.292	-39.0321	13.0321
L4	L1	39.6667*	11.68332	.007**	13.6346	65.6987
	L2	-5.0000	10.44988	.643	-28.2838	18.2838

	L3	9.6667	10.44988	.377	-13.6171	32.9505
	L5	-21.0000	10.44988	.072	-44.2838	2.2838
	adult	-3.3333	11.68332	.781	-29.3654	22.6987
	L1	60.6667*	11.68332	.000**	34.6346	86.6987
	L2	16.0000	10.44988	.157	-7.2838	39.2838
L5	L3	30.6667*	10.44988	.015*	7.3829	53.9505
	L4	21.0000	10.44988	.072	-2.2838	44.2838
	adult	17.6667	11.68332	.161	-8.3654	43.6987
	L1	43.0000*	12.79844	.007**	14.4833	71.5167
	L2	-1.6667	11.68332	.889	-27.6987	24.3654
adult	L3	13.0000	11.68332	.292	-13.0321	39.0321
	L4	3.3333	11.68332	.781	-22.6987	29.3654
	L5	-17.6667	11.68332	.161	-43.6987	8.3654

*** : P≤ 0.05 , ** : P≤ 0.01**

Supplementary Figures

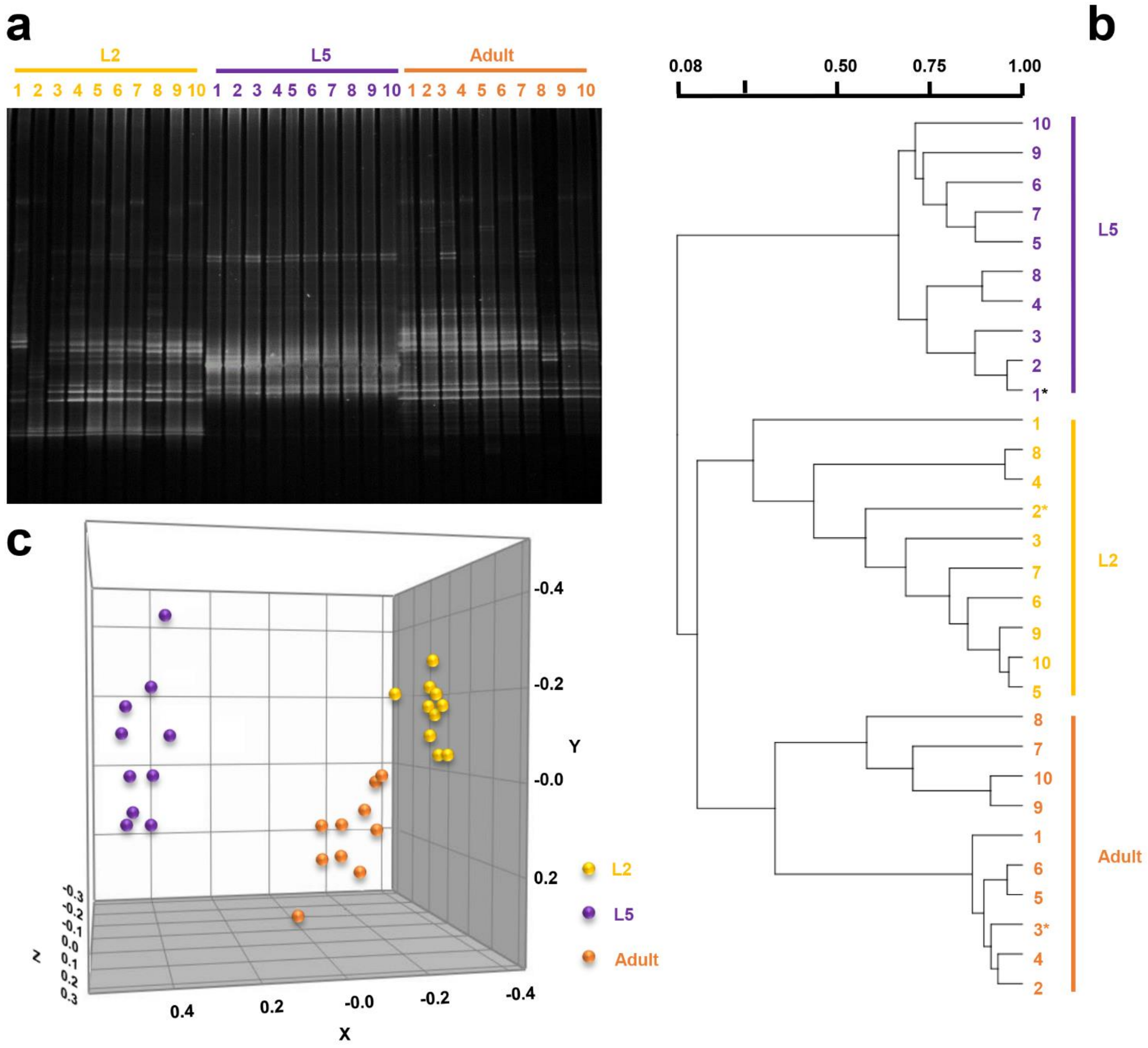

Supplementary Figure S1 DGGE analyses of PCR-amplified 16S rRNA gene fragments of bacterial communities across silkworm life-stage. (a) DGGE patterns of the early-instar (L2) and late-instar (L5) larval gut microbiota and adult gut microbiota of different individuals. (b) Dendrogram of community DGGE fingerprint similarities. Dendrogram was constructed by the unweighted pair group method using arithmetic averages (UPGMA). (c) Multidimensional

scaling (MDS) analysis of the cluster shown in (b). MDS is an optimized 3D representation of the similarity matrix, and these similarities were calculated as a best estimate using the Euclidean distance between two gel lanes (points in the MDS plot) to provide a convenient visual interpretation. The X-, Y-, and Z-axes separately represent three different dimensions: Dim 1, Dim 2, and Dim 3. According to the plot, individual samples (same color key) of each life-stage were grouped together, which suggested the uniqueness and stability of the predominant microbiota composition of each individual. *, represents the MiSeq sequencing sample.

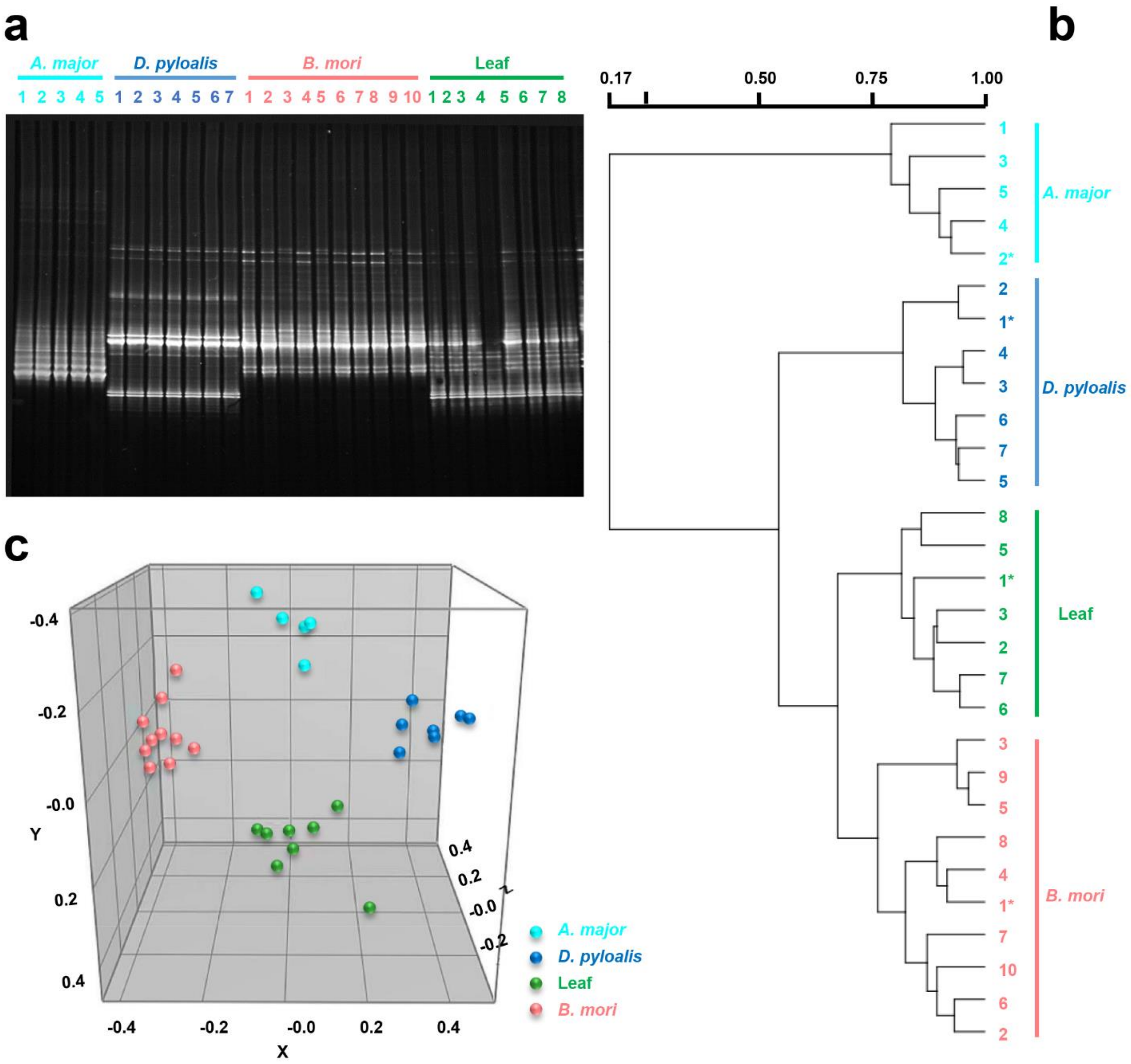

Supplementary Figure S2 DGGE analyses of PCR-amplified 16S rRNA gene fragments of bacterial communities associated with mulberry-feeding Lepidoptera insects and mulberry leaves. (a) DGGE patterns of the final instar larval gut microbiotas of *A. major*, *D. pyloalis* and *B. mori*, and microbiota associated with mulberry leaves. (b) Dendrogram of community DGGE fingerprint similarities. Dendrogram was constructed by the unweighted pair group method using arithmetic averages (UPGMA). (c) Multidimensional scaling (MDS) analysis of the cluster shown in (b). MDS is an optimized 3D

representation of the similarity matrix, and these similarities were calculated as a best estimate using the Euclidean distance between two gel lanes (points in the MDS plot) to provide a convenient visual interpretation. The X-, Y-, and Z-axes separately represent three different dimensions: Dim 1, Dim 2, and Dim 3. According to the plot, individual samples (same color key) of each host species were grouped together, which suggested the uniqueness and stability of the predominant microbiota composition of each individual. *, represents the MiSeq sequencing sample.

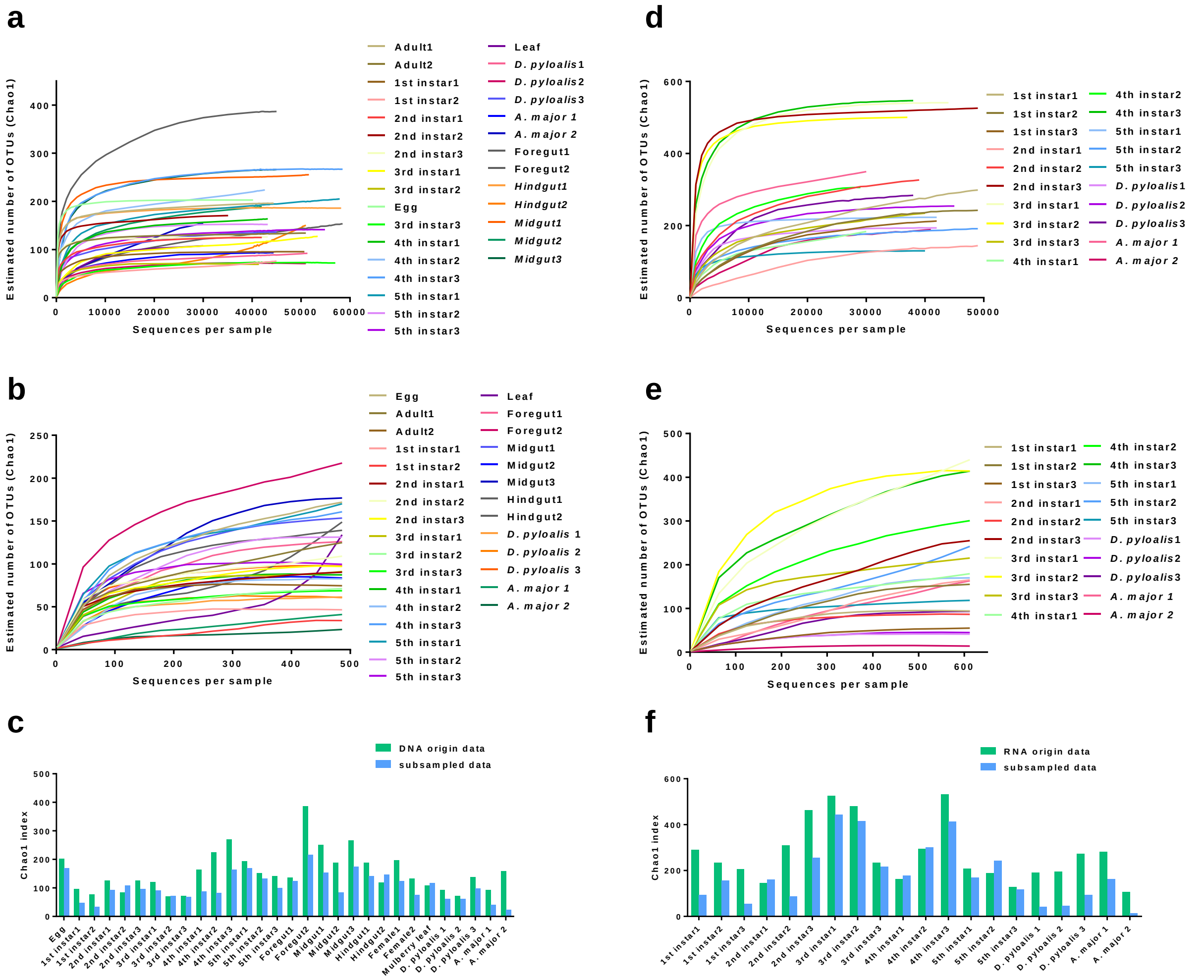

Supplementary Figure S3 Rarefaction curves depicted from original sequencing data sets and randomly subsampled data sets with the same number of 16S sequences. (a) and (d), rarefaction curves of original DNA and RNA sequencing samples. (b) and (e), rarefaction curves of subsampled DNA (486) and RNA (612) sequences. Comparison of species richness between original and subsampled sequencing data was shown in (c) DNA samples and (f) RNA samples. Diversity index was calculated with 1000 iteration.

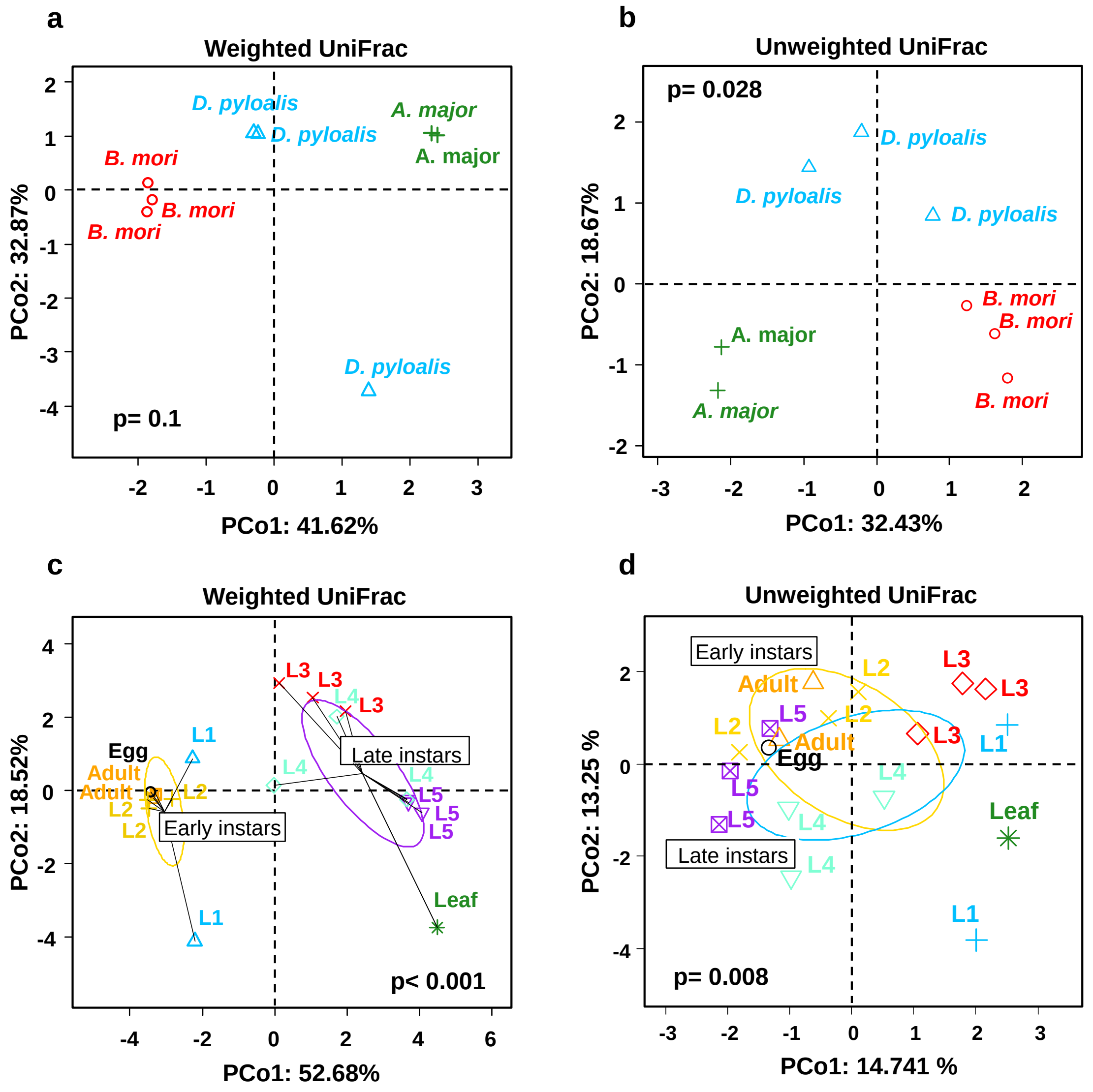

Supplementary Figure S4 PCoA analysis of DNA sequencing samples. (a) PCoA plot using weighted and (b) unweighted UniFrac distance between Lepidoptera species. (c) PCoA plot using weighted and (d) unweighted UniFrac distance across *B. mori* life-stage. PERMANOVA was used for significance analysis, permutation=999.

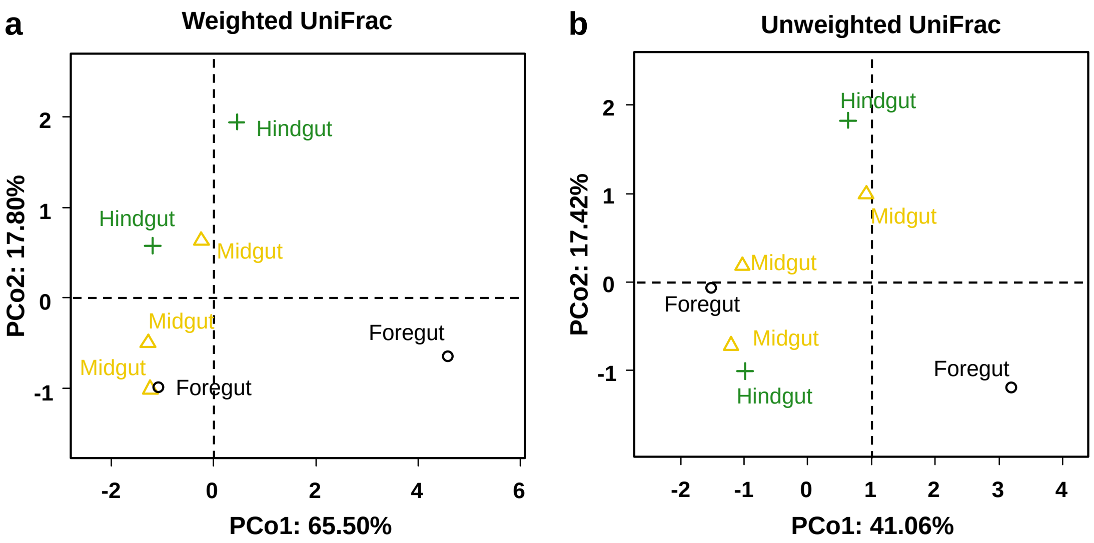

Supplementary Figure S5 PCoA analysis of gut regions. (a) PCoA plot using weighted and (b) unweighted UniFrac distance between gut regions. PERMANOVA was used for significance analysis, permutation=999, P>0.05.

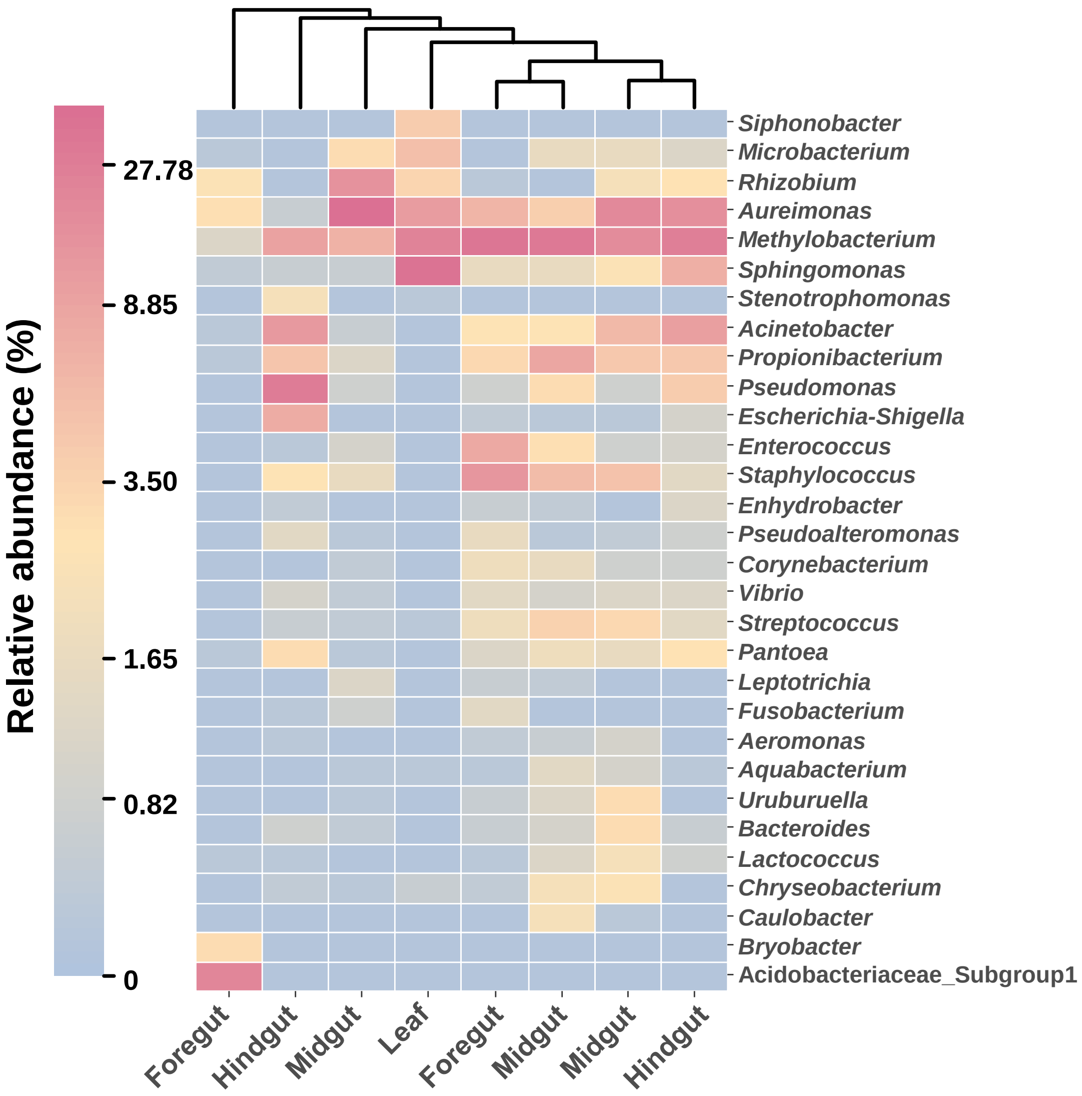

Supplementary Figure S6 Clustering analysis of dominant gut bacteria in different gut regions of the 5th-instar *B. mori*. Relative abundances of the 30 most abundant genera are shown in a heatmap, with cluster analysis using Bray-Curtis distance, followed by a complete-linkage method.

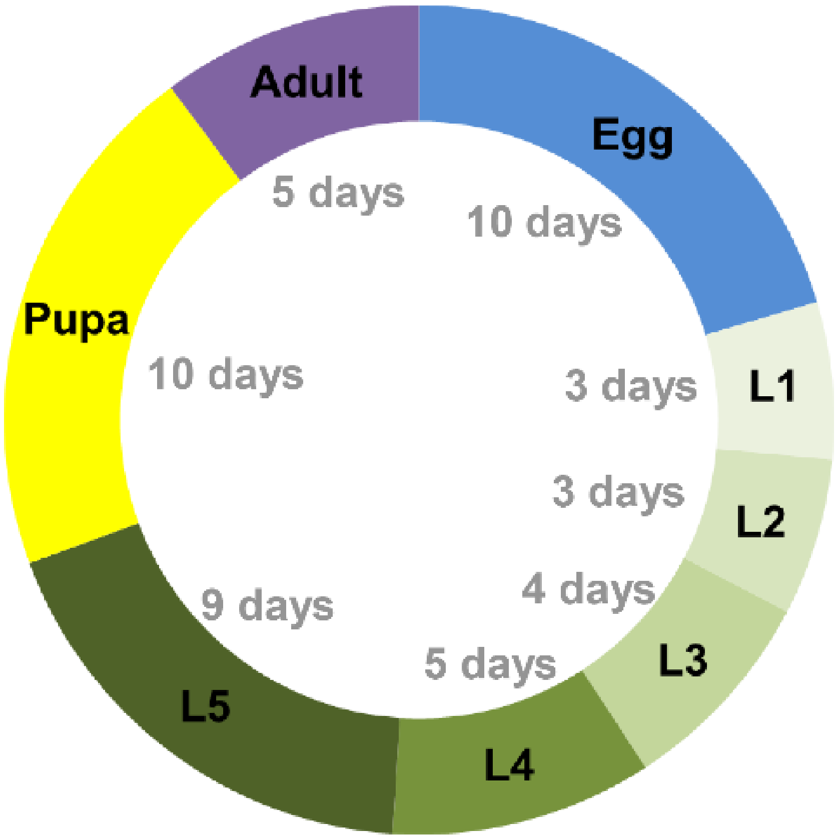

Supplementary Figure S7 *Bombyx mori* life cycle.

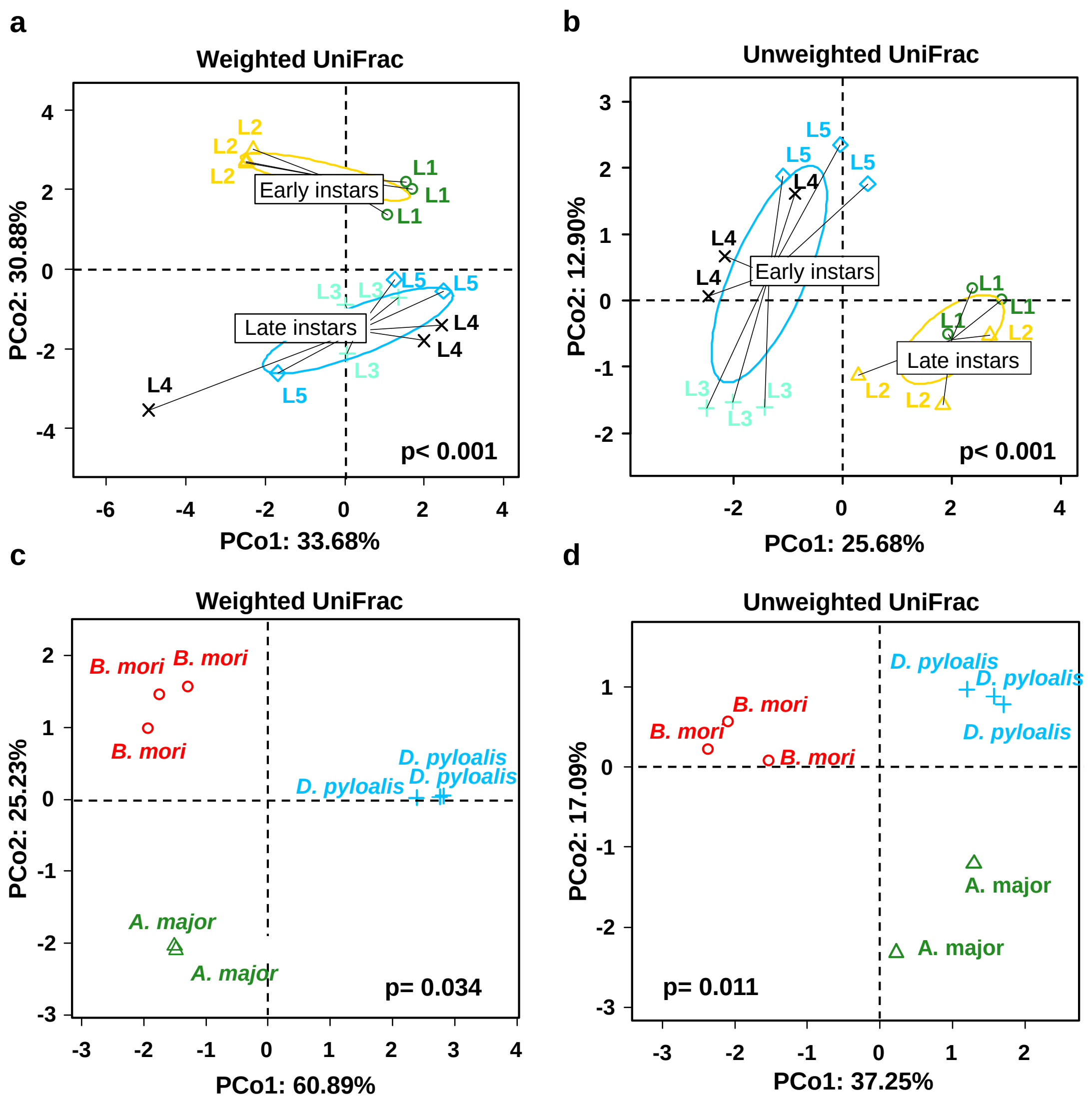

Supplementary Figure S8 PCoA analysis of RNA sequencing samples. (a) PCoA plot using weighted and (b) unweighted UniFrac distance across *B. mori* life-stage. (c) PCoA plot using weighted and (d) unweighted UniFrac distance between Lepidoptera species. PERMANOVA was used for significance analysis, permutation=999.

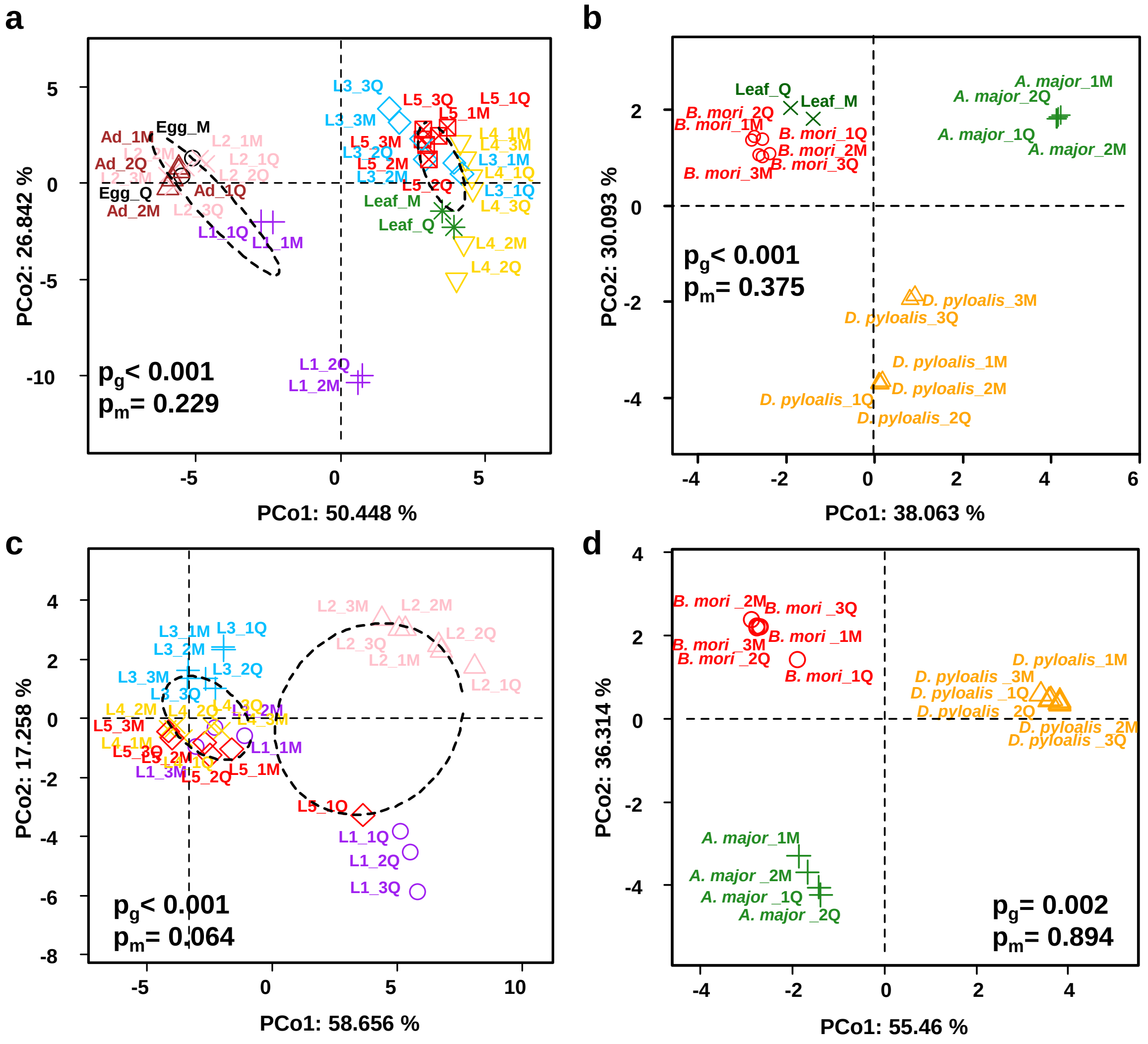

Supplementary Figure S9 Comparison of sequencing results analyzed by mothur and by QIIME. (a) PCoA plot using Bray–Curtis distances across *B. mori* life-stage and (b) between Lepidoptera species in DNA sequencing samples. (c) PCoA plot using Bray–Curtis distances across *B. mori* life-stage and (d) between Lepidoptera species in RNA sequencing samples. Bray-Curtis matrix was generated from the OTU table combining the 30 most abundant genera from QIIME and mothur, respectively. M, represents data extracted from mothur; Q, from QIIME. P value, calculated by PERMANOVA at 999 permutation. Pg, P

value of group variation between life-stage (a, c) or host species (b, d). Pm, P value of method variation between mothur and QIIME analysis.

References

Anderson IC, Cairney JW (2004). Diversity and ecology of soil fungal communities: increased understanding through the application of molecular techniques. *Environ Microbiol* **6**: 769-779.

Bolger AM, Lohse M, Usadel B (2014). Trimmomatic: a flexible trimmer for Illumina sequence data. *Bioinformatics* **30**: 2114-2120.

Caporaso JG, Kuczynski J, Stombaugh J, Bittinger K, Bushman FD, Costello EK *et al.* (2010). QIIME allows analysis of high-throughput community sequencing data. *Nat Methods* **7**: 335-336.

Chen B, Teh BS, Sun C, Hu S, Lu X, Boland W *et al.* (2016). Biodiversity and activity of the gut microbiota across the life history of the insect herbivore *Spodoptera littoralis*. *Sci Rep* **6**: 29505.

DeSantis TZ, Hugenholtz P, Larsen N, Rojas M, Brodie EL, Keller K *et al.* (2006). Greengenes, a chimera-checked 16S rRNA gene database and workbench compatible with ARB. *Appl Environ Microbiol* **72**: 5069-5072.

Edgar RC (2010). Search and clustering orders of magnitude faster than BLAST. *Bioinformatics* **26**: 2460-2461.

Egert M, Stingl U, Bruun LD, Pommerenke B, Brune A, Friedrich MW (2005). Structure and topology of microbial communities in the major gut compartments of *Melolontha melolontha* larvae (Coleoptera: Scarabaeidae). *Appl Environ Microbiol* **71**: 4556-4566.

Gilleland E, Viii G (2014). R Development Core Team (2008). R: A language and environment for statistical computing. R Foundation for Statistical Computing.

Kelly BJ, Gross R, Bittinger K, Sherrill-Mix S, Lewis JD, Collman RG *et al.* (2015). Power and sample-size estimation for microbiome studies

using pairwise distances and PERMANOVA. *Bioinformatics* **31**: 2461-2468.

Koljalg U, Nilsson RH, Abarenkov K, Tedersoo L, Taylor AF, Bahram M *et al.* (2013). Towards a unified paradigm for sequence-based identification of fungi. *Mol Ecol* **22**: 5271-5277.

Lee DH, Zo YG, Kim SJ (1996). Nonradioactive method to study genetic profiles of natural bacterial communities by PCR-single-strand-conformation polymorphism. *Appl Environ Microbiol* **62**: 3112-3120.

Lu H, He J, Wu Z, Xu W, Zhang H, Ye P *et al.* (2013). Assessment of microbiome variation during the perioperative period in liver transplant patients: a retrospective analysis. *Microbial Ecol* **65**: 781-791.

Magoc T, Salzberg SL (2011). FLASH: fast length adjustment of short reads to improve genome assemblies. *Bioinformatics* **27**: 2957-2963.

McDonald D, Price MN, Goodrich J, Nawrocki EP, DeSantis TZ, Probst A *et al.* (2012). An improved Greengenes taxonomy with explicit ranks for ecological and evolutionary analyses of bacteria and archaea. *ISME J* **6**: 610-618.

Mukherjee PK, Chandra J, Retuerto M, Sikaroodi M, Brown RE, Jurevic R *et al.* (2014). Oral mycobiome analysis of HIV-infected patients: identification of *Pichia* as an antagonist of opportunistic fungi. *PLoS Pathog* **10**: e1003996.

Oksanen J, Kindt R, Legendre P, O'Hara B, Stevens MHH, Oksanen MJ *et al.* (2007). The vegan package. *Community ecology package* **10**: 631-637.

Price MN, Dehal PS, Arkin AP (2010). FastTree 2--approximately maximum-likelihood trees for large alignments. *PloS One* **5**: e9490.

Pruesse E, Quast C, Knittel K, Fuchs BM, Ludwig W, Peplies J *et al.* (2007). SILVA: a comprehensive online resource for quality checked and aligned ribosomal RNA sequence data compatible with ARB. *Nucleic Acids Res* **35**: 7188-7196.

R (2014). R: A language and environment for statistical computing. Vienna, Austria: R Foundation for Statistical Computing; 2014.

Schloss PD (2008). Evaluating different approaches that test whether microbial communities have the same structure. *ISME J* **2**: 265-275.

Schloss PD, Westcott SL, Ryabin T, Hall JR, Hartmann M, Hollister EB *et al.* (2009). Introducing mothur: open-source, platform-independent, community-supported software for describing and comparing microbial communities. *Appl Environ Microbiol* **75**: 7537-7541.

Shao Y, Arias-Cordero E, Guo H, Bartram S, Boland W (2014). In vivo Pyro-SIP assessing active gut microbiota of the cotton leafworm, *Spodoptera littoralis*. *PLoS One* **9**: e85948.

Takano SI, Tuda M, Takasu K, Furuya N, Imamura Y, Kim S *et al.* (2017). Unique clade of alphaproteobacterial endosymbionts induces complete cytoplasmic incompatibility in the coconut beetle. *Proc Natl Acad Sci U S A* **114**: 6110-6115.

Tang ZZ, Chen G, Alekseyenko AV (2016). PERMANOVA-S: association test for microbial community composition that accommodates confounders and multiple distances. *Bioinformatics* **32**: 2618-2625.

Thompson CL, Wang B, Holmes AJ (2008). The immediate environment during postnatal development has long-term impact on gut community structure in pigs. *ISME J* **2**: 739-748.

Wang Q, Garrity GM, Tiedje JM, Cole JR (2007). Naive Bayesian classifier for rapid assignment of rRNA sequences into the new bacterial taxonomy. *Appl Environ Microbiol* **73**: 5261-5267.

Wickham H (2016). *ggplot2: elegant graphics for data analysis*. Springer.

Xiong J, Liu Y, Lin X, Zhang H, Zeng J, Hou J *et al.* (2012). Geographic distance and pH drive bacterial distribution in alkaline lake sediments across Tibetan Plateau. *Environ Microbiol* **14**: 2457-2466.

Yun JH, Roh SW, Whon TW, Jung MJ, Kim MS, Park DS *et al.* (2014). Insect gut bacterial diversity determined by environmental habitat, diet, developmental stage, and phylogeny of host. *Appl Environ Microbiol* **80**: 5254-5264.
